# *BMC Emergency Medicine* reviewer acknowledgement 2015

**DOI:** 10.1186/s12873-016-0077-2

**Published:** 2016-02-18

**Authors:** Giulia Mangiameli

**Affiliations:** BioMed Central, Floor 6, 236 Gray’s Inn Road, London, WC1X 8HB, United Kingdom

## Abstract

The editors of *BMC Emergency Medicine* would like to thank all our reviewers who have contributed to the journal in Volume 15 (2015)

**Imo Aisiku**

USA

**Samina Ali**

Canada

**Jose Alonso-Iñigo**

Spain

**Dug Andrusiek**

Canada

**Ali Ardalan**

Iran

**Bonnie Arquilla**

USA

**Marc Auerbach**

USA

**Julie Bembenisty**

Israel

**Anthony Bleetman**

UK

**Andrew Boyd**

USA

**Janet Bray**

Australia

**Alexandra Brazinova**

Austria

**Mette Brekke**

Norway

**Jane Brice**

USA

**Georges Brousse**

France

**Lane Burgette**

USA

**Pietro Caironi**

Italy

**Phillipo Leo Chalya**

Tanzania

**Yves Chaput**

Canada

**Mario Chiavarelli**

Italy

**Stanford Chihuri**

USA

**Jeff Clawson**

USA

**Shelley Cox**

Australia

**Christopher Coyne**

USA

**Janet Curtis**

Australia

**Jihong Dai**

China

**Andrew Dawson**

Australia

**Conor Deasy**

Ireland

**Antonio Dell'Anna**

Italy

**Scott Devenish**

Australia

**Esperanza Diaz**

Norway

**Bridget Dicker**

New Zealand

**Pratik Doshi**

USA

**Bin Du**

China

**Jhodie Duncan**

Australia

**Kylie Dyson**

Australia

**Paul Eustace**

Ireland

**Stephanie Fincham-Campbell**

UK

**Gerry Fitzgerald**

Australia

**Roberto Forero**

Australia

**Federico Franchi**

Italy

**Erika Frischknecht Christensen**

Denmark

**Cornelia Genbrugge**

Belgium

**Georgios Giannakopoulos**

Netherlands

**Scott Goldberg**

USA

**Morris Gordon**

UK

**Arthur Greenberg**

USA

**Patrick Henn**

Ireland

**Falco Hietbrink**

Netherlands

**Nico Hoogerwerf**

Netherlands

**Amir Jalali**

Iran

**Anna Jönsson**

Sweden

**John Kalbfleisch**

USA

**Julie Kanter-Washko**

USA

**Kianoush Kashani**

USA

**David Katz**

USA

**Bory Kea**

USA

**Jakub Kenig**

Poland

**Sankalp Khanna**

Australia

**Kosaku Kinoshita**

Japan

**Frederick Kofi Korley**

USA

**Emmanuel Lagarde**

France

**Melissa Langhan**

USA

**Outi Lapatto-Reiniluoto**

Finland

**Andrew Chee Keng Lee**

UK

**Resa Lewiss**

USA

**Li Luo**

China

**Mary Mackesy-Amiti**

USA

**Ibrahim Mahmoud**

Australia

**Marek Majdan**

Slovakia

**Sheryl Martin-Schild**

USA

**Rahim Moineddin**

Canada

**Dan Mullany**

Australia

**Sebbane Mustapha**

France

**Hüseyin Narcı**

Turkey

**Sagar Nigwekar**

USA

**Alexander Olaussen**

Australia

**Don Olson**

USA

**Kent Olson**

USA

**Peter O'Meara**

Australia

**Sean P. Kelly**

USA

**Anna Paldy**

Hungary

**Sukhmeet Panesar**

UK

**Robert Patton**

UK

**Roshini Peiris-John**

New Zealand

**Timothy Platts-Mills**

USA

**Senija Rasic**

Bosnia and Herzegovina

**Joseph Restuccia**

USA

**Joao Rezende-Neto**

Canada

**Zaccaria Ricci**

Italy

**Schalk Richard**

Germany

**Giuseppe Ristagno**

Italy

**Stefano Romagnoli**

Italy

**Martin Rusnak**

Slovakia

**Fabio Salvi**

Italy

**Claudio Sandroni**

Italy

**Dena Schanzer**

Canada

**Patrick Schober**

Netherlands

**Michael Schull**

Canada

**Raghu Seethala**

USA

**Greene Shepherd**

USA

**Lorraine Sheppard**

Australia

**Jordan Simanjuntak**

USA

**Cor Slagt**

Netherlands

**Steve Socransky**

Canada

**Eldar Soreide**

Norway

**Tracy Stecker**

USA

**Amy Stuck**

USA

**Kostas Stylianou**

Greece

**Andrew Tabner**

UK

**Fabio Silvio Taccone**

Belgium

**Benjamin Taylor**

USA

**Lateef Thanni**

Nigeria

**Lizbet Todorova**

Sweden

**Mahmoud Torabi**

Canada

**Vicken Totten**

USA

**Cynthia Van Der Starre**

Netherlands

**Kathleen Ventre**

USA

**Giovanni Volpicelli**

Italy

**Carl Von Baeyer**

Canada

**David Wampler**

USA

**Hao Wang**

China

**Richard Weinert**

USA

**Scott Weinstein**

Australia

**Ben White**

USA

**Michael Wilson**

USA

**Keramettin Yanik**

Turkey

